# Supplementary material for: Identification and functional characterization of the putative members of the CTDK-1 kinase complex as regulators of growth and development in Aspergillus nidulans and Aspergillus fumigatus
Source: mBio. 2023 Nov 9;14(6):e02452-23. doi: 10.1128/mbio.02452-23 (PMC10746219; doi:10.1128/mbio.02452-23)
Supplement: Supplemental figures — Fig. S1 to S7. [file mbio.02452-23-s0002.pdf]

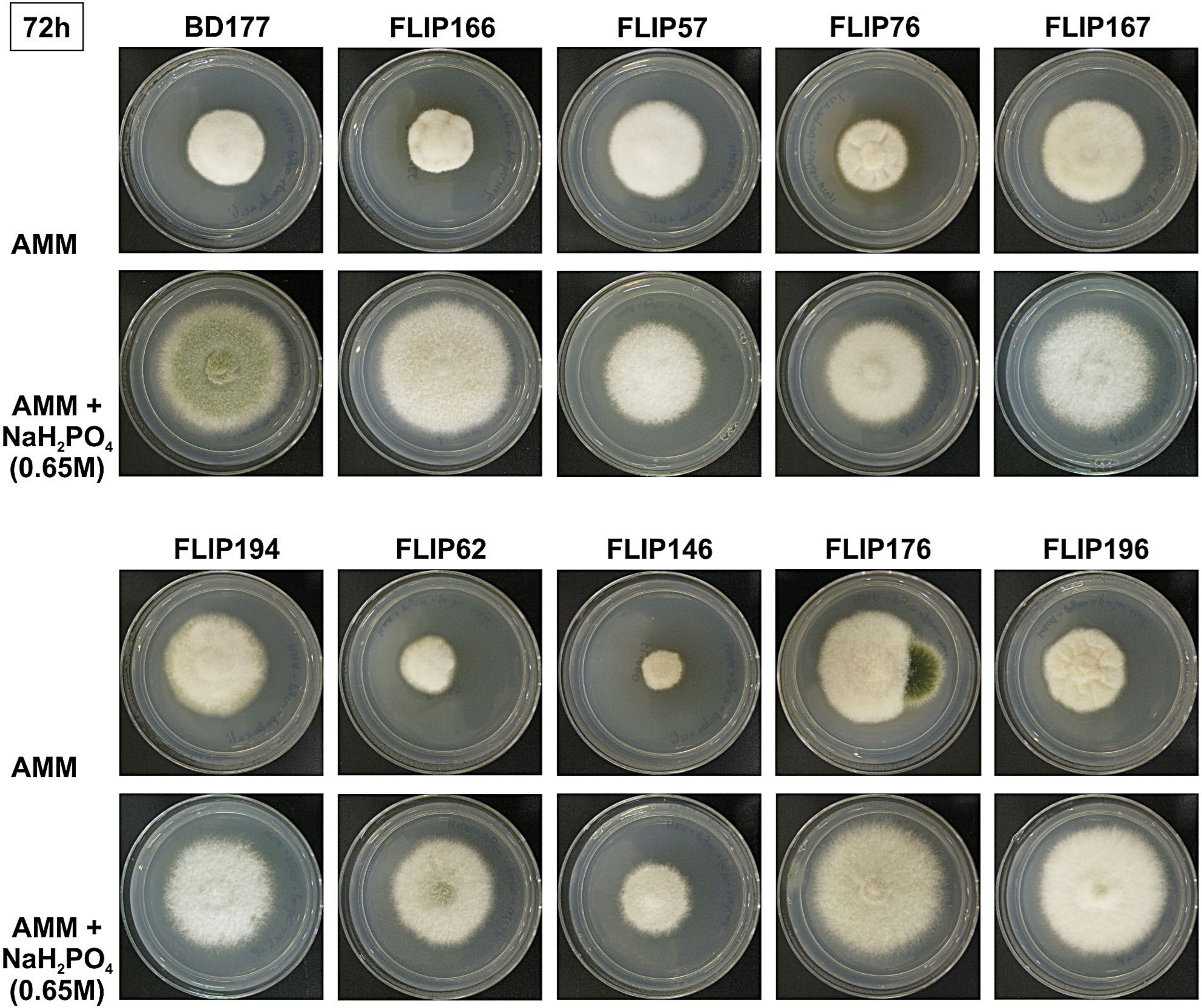
72h
BD177
FLIP166
FLIP57
FLIP76
FLIP167
AMM
AMM + NaH_2PO_4 (0.65M)
FLIP194
FLIP62
FLIP146
FLIP176
FLIP196
AMM
AMM + NaH_2PO_4 (0.65M)

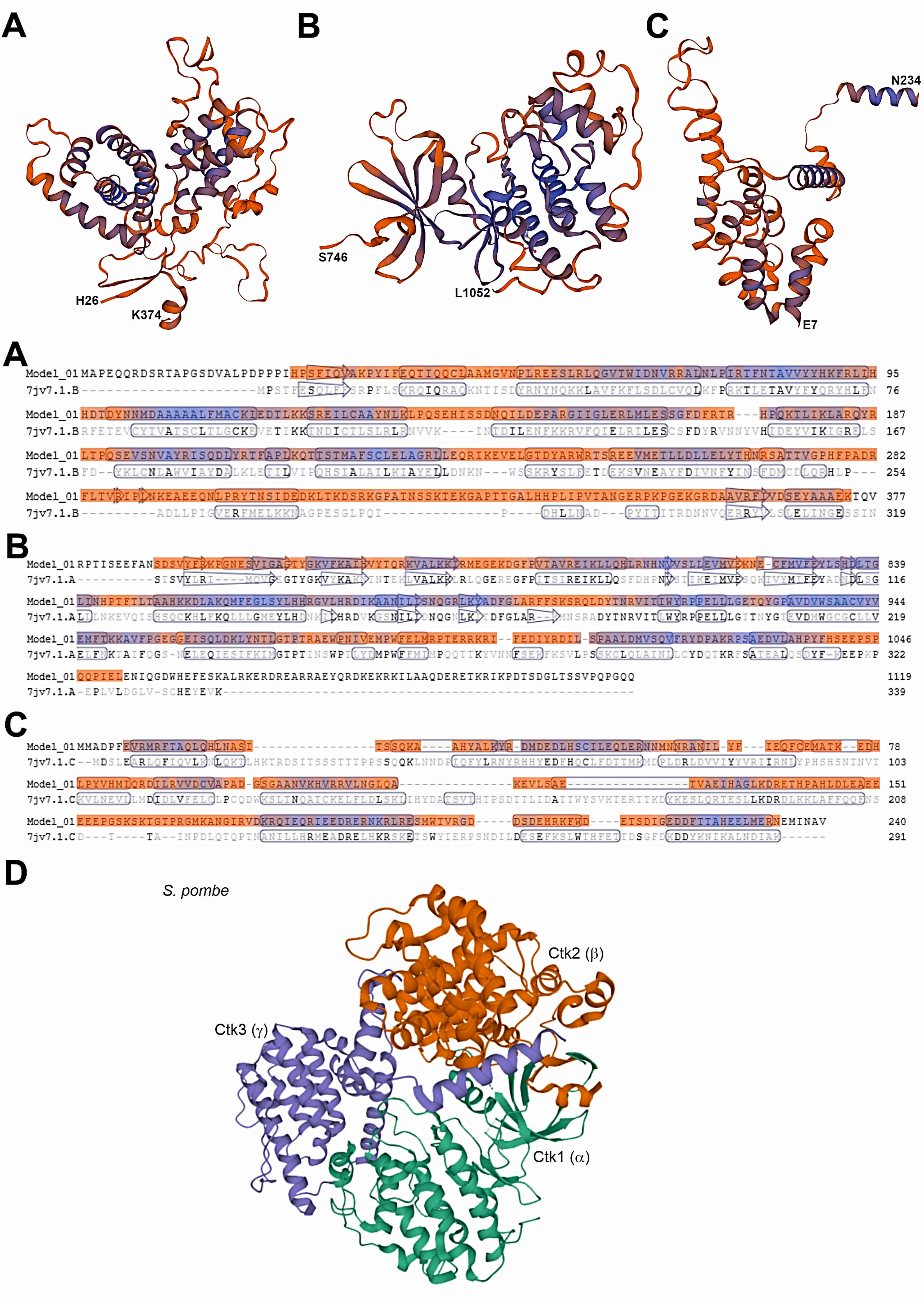

A
H26
K374
B
S746
L1052
C
N234
E7
A
Model_01 MAPEQQRDSRTAPGSDVALPDPPPIHPSFIQVAKPYIFEQTIQQCLAAMGVNPLREESLRLQGVTWIDNVRRALNLPIRTFNTAVVYYHKFRLIH 95
7jv7.1.B ---------------------MPSTFESQLFFSRPFLSKRQIQRAQKNTISDYRNYNQKKLAVFKFLSDLCVQLKFPRKTLETAVYFYQRYHLFN 76
Model_01 HDTDYNNMDAAAAALFMACKIEDTLKKSREILCAAYNLKLPQSEHISSDNQILDEPARGIIGLERLMLESSGFDFRTR---HPQKTLIKLARQYR 187
7jv7.1.B RFETEVCYTVATSCLTLGCKEVETIKKINDICTLSLRLRNVVK----INIDILENFKKRVFQIELRILESCSFDYRVNNYVHIDEYVIKIGRELS 167
Model_01 LTPQSEVSNVAYRISQDLYRTFAPLKQTTSTMAFSCLELAGRLLEQRIKEVELGTDYARWRTSREEVMETLLDLLELYTHNRSATTVGPHFPADR 282
7jv7.1.B FD--YKLCNLAWVIAYDALKLETILVIPQHSIALAILKIAYELLDNKN---WSSKRYSLFETDEKSVNEAYFDIVNFYINSFDMCDLQRHLP--- 254
Model_01 FLTVRIPLNKEAEEQNLPRYTNSIDEDKLTKDSRKGPATNSSKTEKGAPTTGALHHPLIPVTANGERPKPGEKGRDAAVRFIVDSEYAAAEKTQV 377
7jv7.1.B ---------ADLLPIGVERFMELKKNAGPESGLPQI--------------P----DHLLNAD---PYITITRDNNVQERRYVLSLELINGESSIN 319
B
Model_01 RPTISEEFANSDSVYFRKPGNESVIGAGTYGKVFKAIHVYTQRKVALKKIRMEGEKDGFPVTAVREIKLLQHLRNHNVVSLLEVMVEKNE-CFMVFEYLSHDLTG 839
7jv7.1.A ----------STSVYLRI----MQVGEGTYGKVYKAKNINTEKLVALKKLRLQGEREGFPITSIREIKLLQSFDHPNVSTIKEIMVESQKTVYMIFEYADNDLSG 116
Model_01 LINHPTFTLTAAHKKDLAKQMFEGLSYLHHRGVLHRDIKAANILISNQGRLKYADFGLARFFSKSRQLDYTNRVITIWYRPPELLLGETQYGPAVDVWSAACVYV 944
7jv7.1.A LLNKEVQISHSQCKHLFKQLLLGMEYLHDNKILHRDVKGSNILIDNQGNLKIIDFGLAR--MNSRADYTNRVITLWYRPPELLLGTTNYGTEVDMWGCGCLLV 219
Model_01 EMFTKKAVFPGEGGEISQLDKLYNTLGTPTRAEWPNIVEMPWFELMRPTERRKRI--FEDIYRDIL-SPAALDMVSQVFRYDPAKRPSAEDVLAHPYFHSEEPSP 1046
7jv7.1.A ELFNKTAIFQGS-NELEQIESIFKIMGTPTINSWPTLYDMPWFFMIMPQQTTKYVNNFSEKFKSVLPSSKCLQLAINLLCYDQTKRFSATEALQSDYF-EEPKP 322
Model_01 QQPIELENIQGDWHEFESKALRKERDREARRAEYQRDKEKRKILAAQDERETKRIKPDTSDGLTSSVPQPGQQ 1119
7jv7.1.A -EPLVLDGLV-SCHEYEVK-- 339
C
Model_01 MMADPFEVRMRFTAQLQHLNASI----------------TSSQKA----AHYALKYR-DMDEDLHSCILEQLERNNMNNRANIL-YF---IEQFCEMATK--EDH 78
7jv7.1.C --MDSLEARLQFIQVLKNLQKTLHKTRDSITSSSTTTPPSSQQKLNNDPIQFYLRNYRHHYEDFHQCLFDTTMKMDPLDRLDVVIYYVRIIRNIYPHSHSNTNVT 103
Model_01 LPYVHMIQRDILRVVDCVAPAD-GSGAANVKHVRRVLNGLQA----------------KEVLSAE----------------TVAEIHAGLKDRETHPAHLDLEAEE 151
7jv7.1.C KVLNEVILMDIDLVFELCLPCQDWKSLTNQATCKELFLDLSKLIHYDATSVIHTPSDTTLIDATTWYSVKTERTTKDVKESLQRTESLLKDRDLKKLAFFQQFNS 208
Model_01 EEEPGSKSKTGTPRGMKANGIRVDKRQIEQRIEEDRERNKRLRESMWTVRGD-----DSDEHRKFWD----ETSDIGEDDFTTAHEELMERNEMINAV 240
7jv7.1.C D---T----TA--INPDLQTQPTNANILLHRMEADRELHKRSKETSWYIERPSNDILDESEFKSLWTHFETIDSGFDKDDYKNIKALNDIAK------ 291
D
S. pombe
Ctk2 (β)
Ctk3 (γ)
Ctk1 (α)

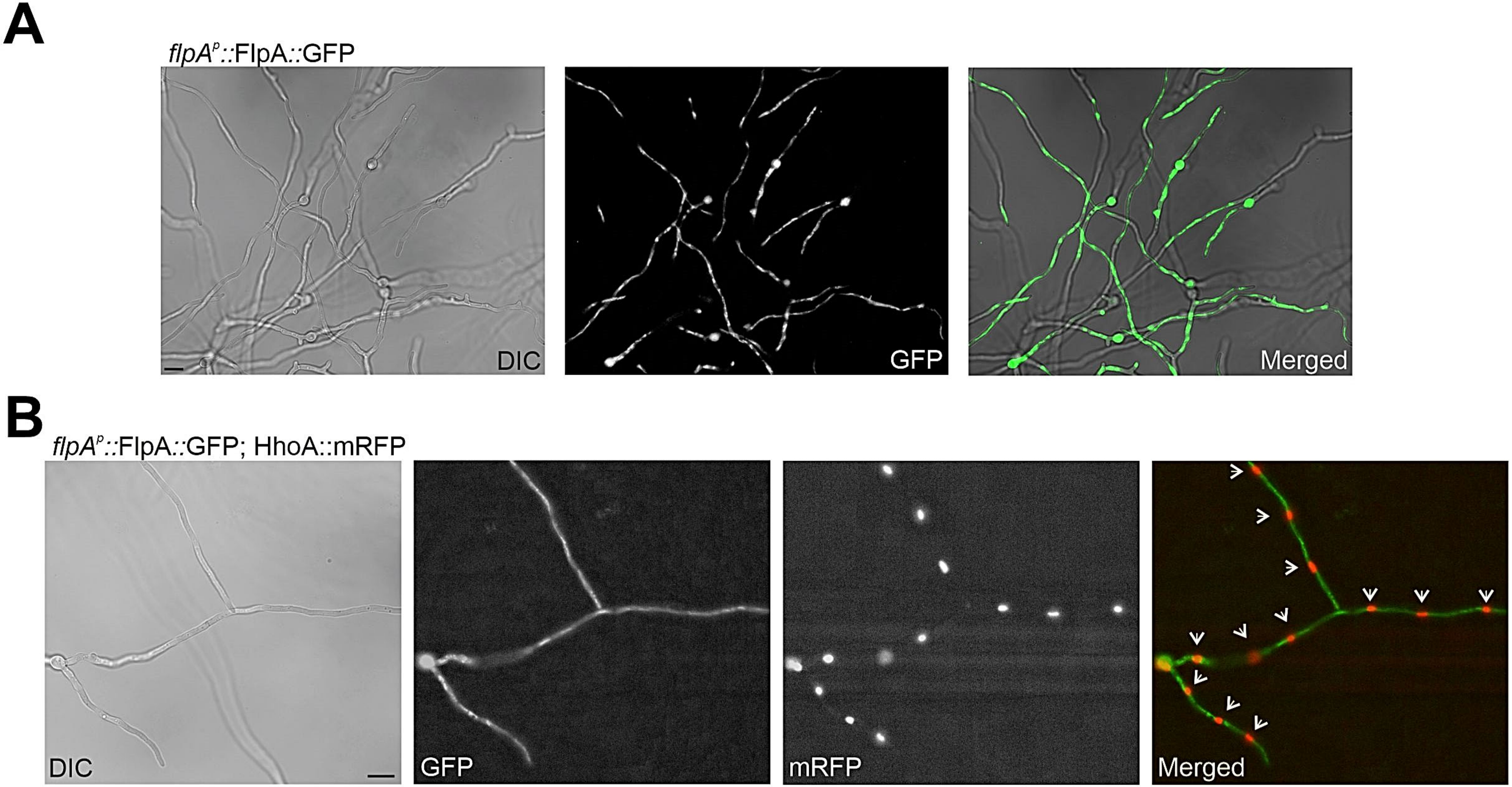
A
flpA^p::FlpA::GFP
DIC
GFP
Merged
B
flpA^p::FlpA::GFP; HhoA::mRFP
DIC
GFP
mRFP
Merged

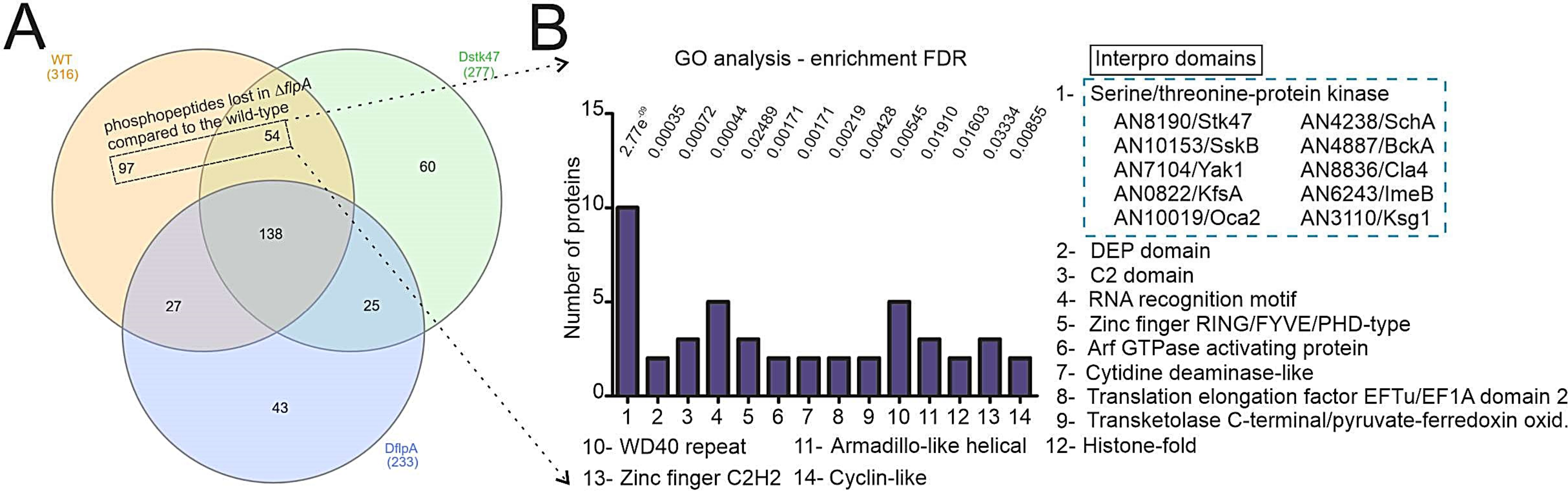
A
WT (316)
Dstk47 (277)
DflpA (233)
phosphopeptides lost in ΔflpA compared to the wild-type
97
54
60
138
27
25
43
B
GO analysis - enrichment FDR
Number of proteins
2.77e-09
0.00035
0.00072
0.00044
0.02489
0.00171
0.00171
0.00219
0.00428
0.00545
0.01910
0.01603
0.03334
0.00855
Interpro domains
1- Serine/threonine-protein kinase
AN8190/Stk47 AN4238/SchA
AN10153/SskB AN4887/BckA
AN7104/Yak1 AN8836/Cla4
AN0822/KfsA AN6243/ImeB
AN10019/Oca2 AN3110/Ksg1
2- DEP domain
3- C2 domain
4- RNA recognition motif
5- Zinc finger RING/FYVE/PHD-type
6- Arf GTPase activating protein
7- Cytidine deaminase-like
8- Translation elongation factor EFTu/EF1A domain 2
9- Transketolase C-terminal/pyruvate-ferredoxin oxid.
10- WD40 repeat
11- Armadillo-like helical
12- Histone-fold
13- Zinc finger C2H2
14- Cyclin-like

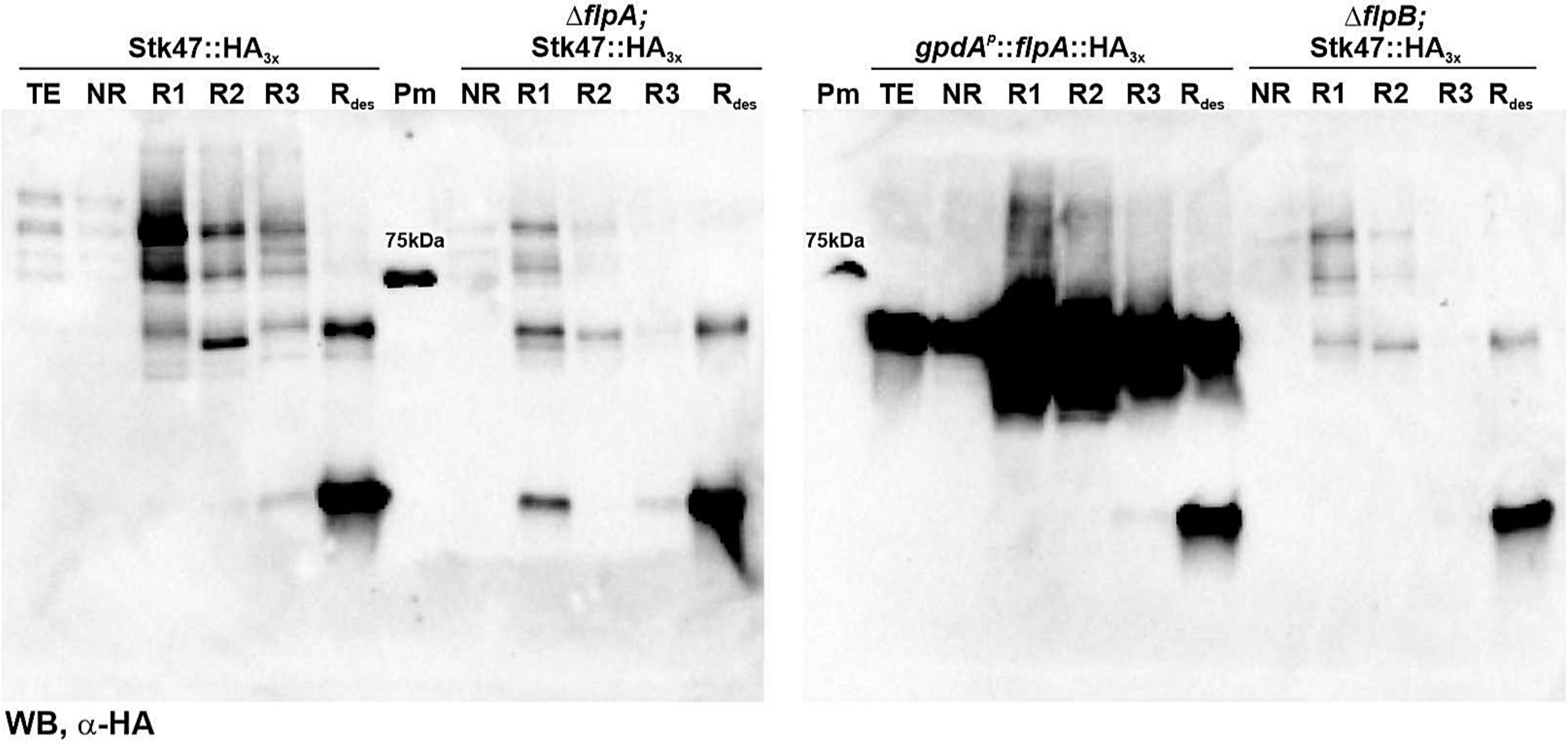

ΔflpA;
Stk47::HA3x
gpdAP::flpA::HA3x
ΔflpB;
Stk47::HA3x
TE NR R1 R2 R3 Rdes Pm NR R1 R2 R3 Rdes
Pm TE NR R1 R2 R3 Rdes NR R1 R2 R3 Rdes
75kDa
75kDa
WB, α-HA

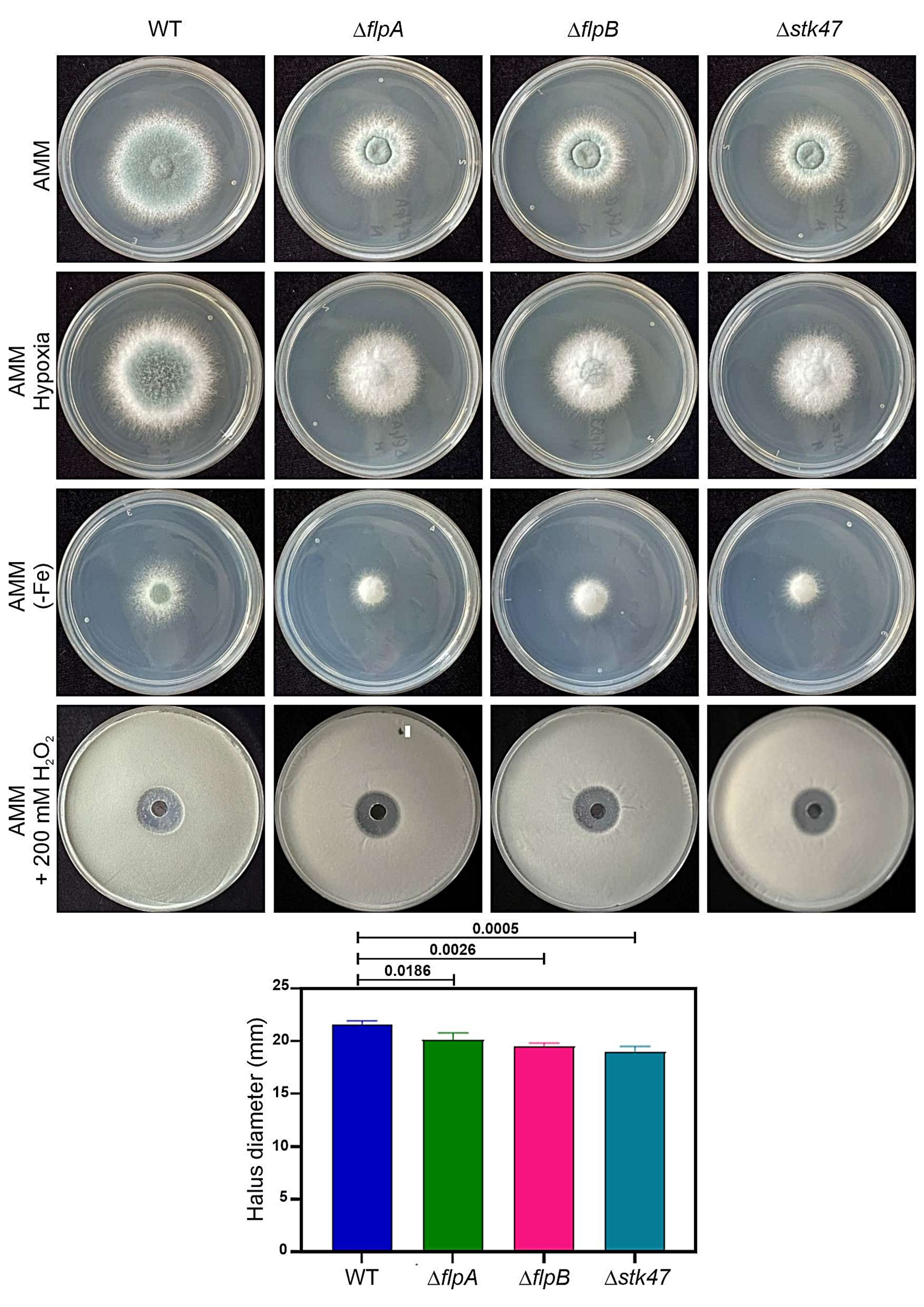
WT
ΔflpA
ΔflpB
Δstk47
AMM
AMM Hypoxia
AMM (-Fe)
AMM + 200 mM H_2O_2
0.0005
0.0026
0.0186
Halus diameter (mm)
25
20
15
10
5
0
WT
ΔflpA
ΔflpB
Δstk47

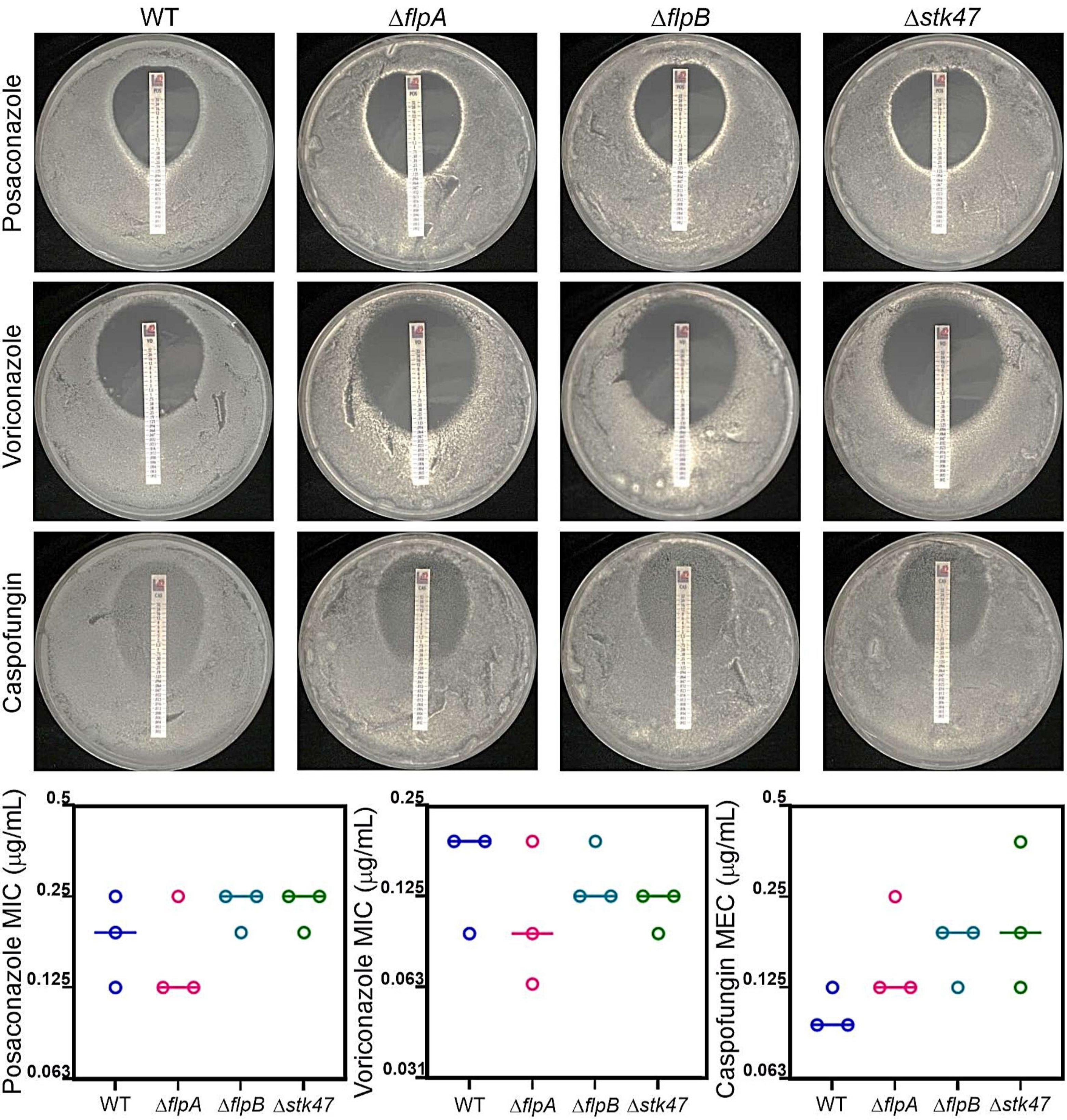
WT
ΔflpA
ΔflpB
Δstk47
Posaconazole
Voriconazole
Caspofungin
Posaconazole MIC (μg/mL)
0.5
0.25
0.125
0.063
WT
ΔflpA
ΔflpB
Δstk47
Voriconazole MIC (μg/mL)
0.25
0.125
0.063
0.031
WT
ΔflpA
ΔflpB
Δstk47
Caspofungin MEC (μg/mL)
0.5
0.25
0.125
0.063
WT
ΔflpA
ΔflpB
Δstk47
