# Supplementary material for: Identification and functional characterization of the putative members of the CTDK-1 kinase complex as regulators of growth and development in Aspergillus nidulans and Aspergillus fumigatus
Source: mBio. 2023 Nov 9;14(6):e02452-23. doi: 10.1128/mbio.02452-23 (PMC10746219; doi:10.1128/mbio.02452-23)
Supplement: Supplemental legends — Legends for supplemental figures and tables. [file mbio.02452-23-s0003.docx]

**Supplementary material.**

**S1 Table. Probable FlpA, Stk47 and FlpB orthologs identified by BLAST analyses.** The table shows the accession number of each sequence, the taxonomy of the species each sequence belongs to, the length as well as score, expect and coverage values. It also shows the results (first hit) of the confirmatory reverse retrieval of each sequence against the *Aspergillus nidulans* proteome in the AspGD database. The color key used is shown on the right of the table and applies to classes of fungi.

**S2 Table. List of phosphopeptides and phosphorylated proteins identified in crude protein extracts of the wild-type and the null *flpA* or null *stk47* strains.**

**S3 Table. Strains used in this work.**

**S4 Table. Oligonucleotides used in this study.**

**S1 File. Candidate exonic mutations found in FLIP57 and FLIP76.** Exonic mutations in FLIP57 (track 1) and FLIP76 (track 2) genomes are shown in comparison to the FGSCA4 reference genome, FLIP166 genome (track 3) or the transcriptomes of wild-type (track 4) or Δ*flbB* (track 5) strains (24, 30, 33).

**S1 Figure. Phenotypes of selected FLIP mutants.** Images were taken after 72 hours of culture at 37 ºC on AMM (row 1) or AMM supplemented with 0.65 M NaH_2_PO_4_ (row 2). Parental Δ*flbB* and FLIP166 mutants were used as controls. Diameter of plates: 5.5 cm.

**S2 Figure. Predicted three-dimensional structures of FlpA (A), Stk47 (B) and FlpB (C), modelled by Swiss-Model.** The models cover the regions His26-Lys374 of FlpA, Ser746-Leu1052 in the case of Stk47 and Glu7-Asn234 in the case of FlpB, and are based on PDB structure 7jv7.1 (37), which can be seen in panel D. The alignments between query sequences and the reference structure can be seen below Swiss-Models.

**S3 Figure: Subcellular localization of FlpA::GFP** when it is expressed driven by its native promoter, in vegetative of A) a wild-type strain, or B) an HhoA::mCherry strain. Scale bars = 5 µm.

**S4 Figure: Phosphopeptide-enrichment analysis for *A. nidulans* wild-type, Δ*flpA* and Δ*stk47* strain**. A) Venn diagram showing the number of phosphorylated proteins detected in wild-type, Δ*flpA* and Δ*stk47* strains. The subgroups of phosphoproteins not detected in the null *flpA* background are highlighted. B) Bar-graph showing the most represented InterPro functions in the list of phosphoproteins not detected in the null *flpA* background compared to the parental wild-type strain. AN8190/Stk47 is included among the ten serine/threonine-protein kinases lost in the Δ*flpA* strain.

**S5 Figure: Enrichment of Stk47::HA_3x_ or FlpA:: HA_3x_ using HA-agarose resin (Pierce).** Crude protein extracts of strains expressing Stk47::HA_3x_ (wild-type, Δ*flpA* or Δ*flpB* genetic backgrounds) or FlpA::HA_3x_ (wild-type background) chimeras were incubated with the resin. Retained fractions were sequentially eluted using non-reducing Laemli buffer (R1, R2 and R3) or SDS-PAGE loading buffer (Rdes). See Materials and Methods. LC-MS/MS analyses of those R fractions did not show significant enrichment in peptides corresponding to putative interactors (FlpA, Stk47 and FlpB) or any other *A. nidulans* cyclin and/or kinases (not shown).

**S6 Figure: Phenotype of *A. fumigatus* single-null mutants of *flpA*, *flpB* and *stk47*** on AMM medium, under oxygen- or iron-limiting conditions, or under oxidative stress, after 48 hours of growth at 37 ºC. Growth inhibition halos were measured after 48 hours.

**S7 Figure: Susceptibility of single-null *flpA*, *flpB* and *stk47* strains of *A. fumigatus* to posaconazole, voriconazole and caspofungin**, determined using gradient diffusion strips in RPMI agar plates. Pictures correspond to 48 hours of growth at 37 ºC.
